# The role of notch signaling in tumor angiogenesis in hematological malignancies

**DOI:** 10.3389/fonc.2026.1904760

**Published:** 2026-08-17

**Authors:** Roberto Tamma, Vincenzo Benagiano, Domenico Ribatti

**Affiliations:** Department of Translational Biomedicine and Neuroscience, University of Bari Medical School, Bari, Italy

**Keywords:** B-cell malignancies, notch signaling pathway, PEST domain mutations, targeted therapies, tumor angiogenesis

## Abstract

The evolutionarily conserved Notch signaling pathway is a contact-dependent communication mechanism regulating cell fate, tissue patterning, and cellular metabolism. Beyond canonical NICD-RBPJ transcription, Notch engages in non-canonical crosstalk with networks like Wnt and NF-κB. In tumor angiogenesis, the endothelial Foxc1/2-DLL4-Notch1 axis coordinates tip/stalk cell plasticity, while restricting glycolytic flux via PFKFB3 downregulation to promote vessel maturation. Consequently, pathway inhibition triggers non-productive hypersprouting and vascular collapse, whereas Notch activation strategies offer potential for therapeutic vessel normalization. Crucially, Notch acts as a driving oncogene in B-cell malignancies, promoting survival and chemoresistance. In mantle cell lymphoma (MCL), somatic mutations truncate the C-terminal PEST domain, preventing proteasomal degradation and inducing constitutive NICD hyperactivation—defining an aggressive, microenvironment-sustained clinical subset. Similar NOTCH1/2 aberrations drive clonal progression in marginal zone and MALT lymphomas, and indicate poor prognosis in Hepatitis C Virus-positive diffuse large B-cell lymphoma (DLBCL). Routine genomic screening for Notch alterations provides critical prognostic stratification, laying the basis for personalized, selective therapies in refractory hematological diseases.

## Introduction

The Notch signaling pathway is an evolutionarily highly conserved, short-range intercellular communication mechanism that governs pivotal biological processes, including cell fate determination, lineage commitment, architectural tissue patterning, and homeostatic maintenance (1). Unlike many other signal transduction cascades that rely on soluble, diffusible morphogens or growth factors, the Notch system operates through physical, mechanical interactions between contiguous cells expressing complementary transmembrane surface proteins (2).

## Notch signaling pathway

### Canonical signaling mechanics

**Figure 1:**
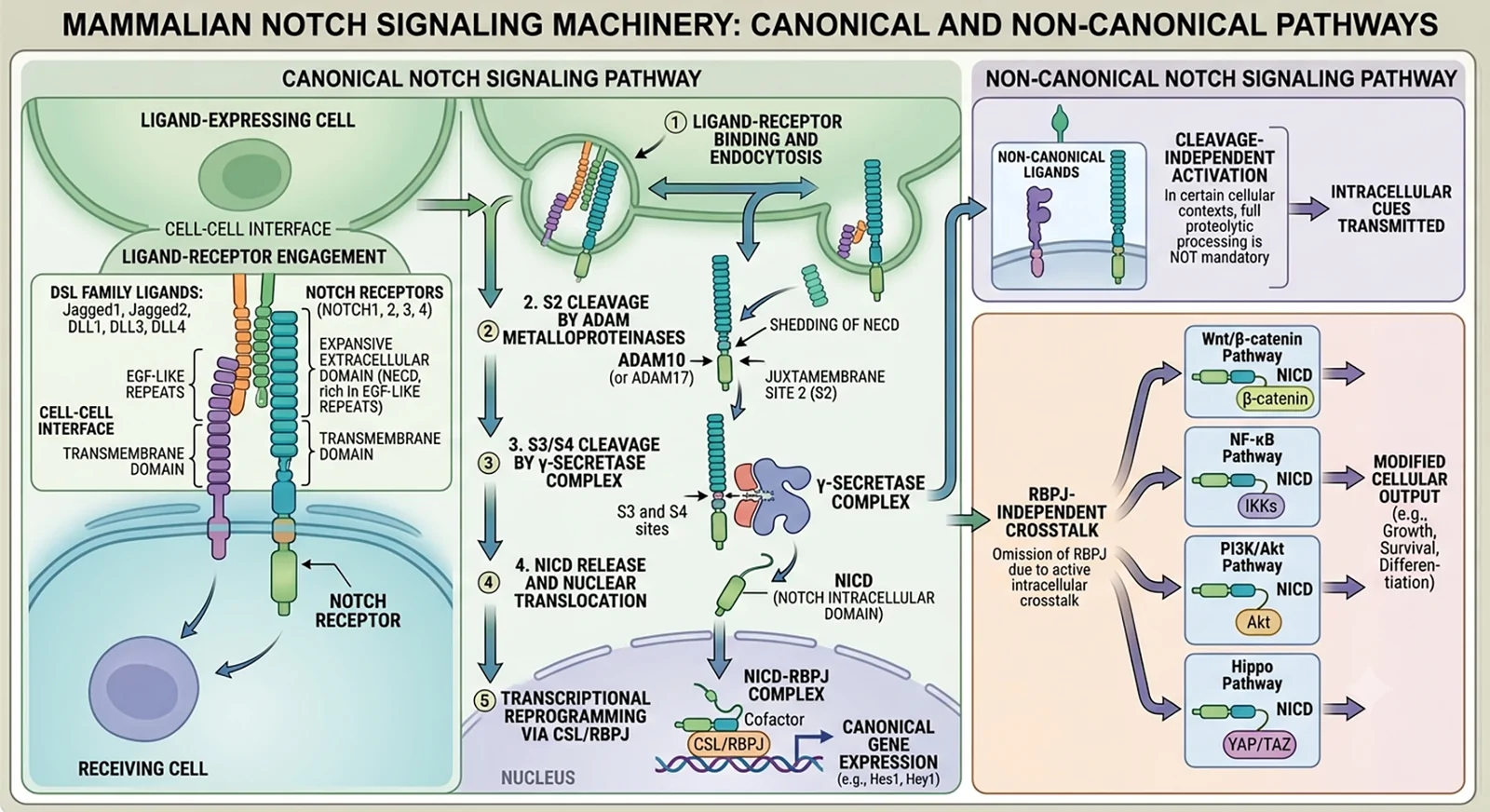
Overview of canonical and non-canonical mammalian notch signaling. canonical pathway (left): Ligand-receptor binding triggers sequential proteolytic cleavages by ADAM metalloproteinases (S2) and the gamma-secretase complex (S3/S4), releasing the Notch Intracellular Domain (NICD) to translocate to the nucleus and drive RBPJ-dependent gene expression. Non-canonical pathways (right): Involve cleavage-independent activation or RBPJ-independent crosstalk, where NICD directly interacts with other major signaling cascades (Wnt, NF-kappaB, PI3K/Akt, Hippo) to alter cellular outcomes.

In mammals, the core machinery comprises four distinct single-pass transmembrane Receptors, Notch1, Notch2, Notch3, and Notch4, and five structured transmembrane ligands belonging to the Delta-Serrate-Lag2 (DSL) family: Jagged1, Jagged2, Delta-like ligand 1 (DLL1), DLL3, and DLL4 (3) (**Figure 1**). Both receptors and ligands are complex glycoproteins featuring expansive extracellular domains rich in epidermal growth factor (EGF)-like repeats, which mediate physical binding selectivity (4).

The canonical Notch signaling pathway is activated when a Notch receptor binds to its ligand on an adjacent cell (DSL family). Ligand binding induces endocytosis of Notch receptor/Ligand complex leading to the exposure of the S2 site on NOTCH and triggers a series of proteolytic cleavages. First, ADAM metalloproteinases, primarily ADAM10, cleave the receptor at the juxtamembrane S2 site, resulting in shedding of the Notch extracellular domain (NECD). The remaining membrane-bound fragment is then cleaved at the S3/S4 sites by the γ-secretase complex, releasing the Notch intracellular domain (NICD). The NICD subsequently translocates to the nucleus, where it interacts with the CSL/recombination signal-binding protein for the immunoglobulin kappa J region (RBPJ) transcriptional complex to regulate the expression of Notch target genes (5, 6) (**Figure 1**).

### Non-canonical (RBPJ-independent) pathways and signaling controversies

While the canonical NICD-RBPJ axis represents the predominant cascade, non-canonical (RBPJ-independent) Notch signaling pathways have been identified (7). These variants display unique characteristics: Cleavage-Independent Activation: In certain cellular contexts initiated by non-canonical ligands, full receptor proteolytic processing may not be strictly mandatory to transmit intracellular cues: RBPJ Independent crosstalk: The omission of RBPJ occurs due to active intracellular crosstalk with parallel signaling networks located upstream or independent of the NICD-RBPJ complex. In these instances, membrane-bound Notch or free NICD interacts directly with other established signaling cascades, such as the Wnt/β-catenin, NF-κB, PI3K/Akt, or Hippo pathways, modifying cellular output without forming the classical CSL nuclear transcription assembly (8) (**Figure 1**).

### Notch in tumor angiogenesis and endothelial metabolism

**Figure 2:**
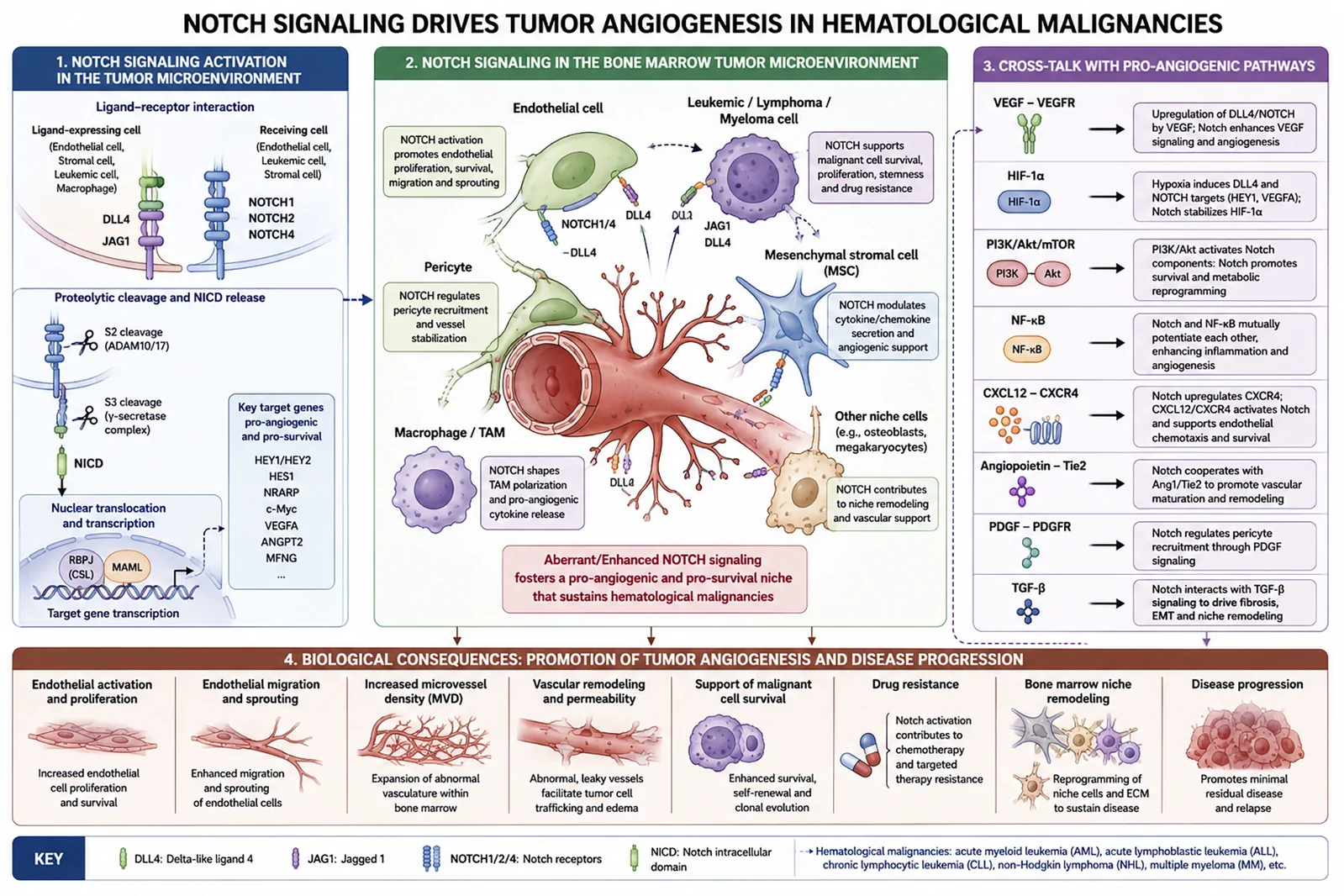
Schematic overview of Notch signaling in tumor angiogenesis in hematological malignancies. Notch signaling integrates interactions between malignant hematopoietic cells and the bone marrow vascular niche, cooperating with major pro-angiogenic pathways to promote endothelial activation, vascular remodeling, tumor cell survival, and disease progression.

Notch signaling coordinates bidirectional communication between malignant hematopoietic cells and the bone marrow vascular niche, integrating endothelial activation, stromal cell interactions, and multiple pro-angiogenic signaling pathways that collectively sustain tumor angiogenesis and disease progression in hematological malignancies (**Figure 2**).

#### Molecular crosstalk of NOTCH with other pathways

Strong VEGFR2 activation triggers downstream cascades that upregulate the expression of the specific Forkhead box transcription factors Foxc1 and Foxc2. Foxc1 and Foxc2 migrate to the nucleus and directly bind specific Dll4 promoter enhancer element or physically disassemble local repressive transcriptional complexes, driving robust expression of the DLL4 ligand on the surface of the tip cell (9, 10).

The high concentration of surface-bound DLL4 on the tip cell physically engages Notch1 receptors expressed on immediate neighboring endothelial cells, triggering the canonical proteolytic cleavage (ADAM10 and γ-secretase) within these adjacent follower cells, releasing NICD. The newly formed nuclear NICD-RBPJ complex directly represses the transcription of VEGFR2 and VEGFR3, downregulating their expression (11, 12).

While DLL4 acts to restrict the tip cell phenotype, the alternative ligand Jagged1 (Jag1) is preferentially and strongly expressed within the stalk cell compartment. Jag1 serves as an endogenous competitive antagonist to DLL4 at the active sprouting front. When Jag1 binds to Notch1 on adjacent cells, it induces a distinct, significantly weaker downstream transcriptional signal compared to DLL4. By displacing DLL4 and dampening excessive Notch1 activation, Jag1 maintains the necessary plasticity and functional equilibrium between tip and stalk cells, allowing new sprouts to form when biologically required (13).

**Figure 3:**
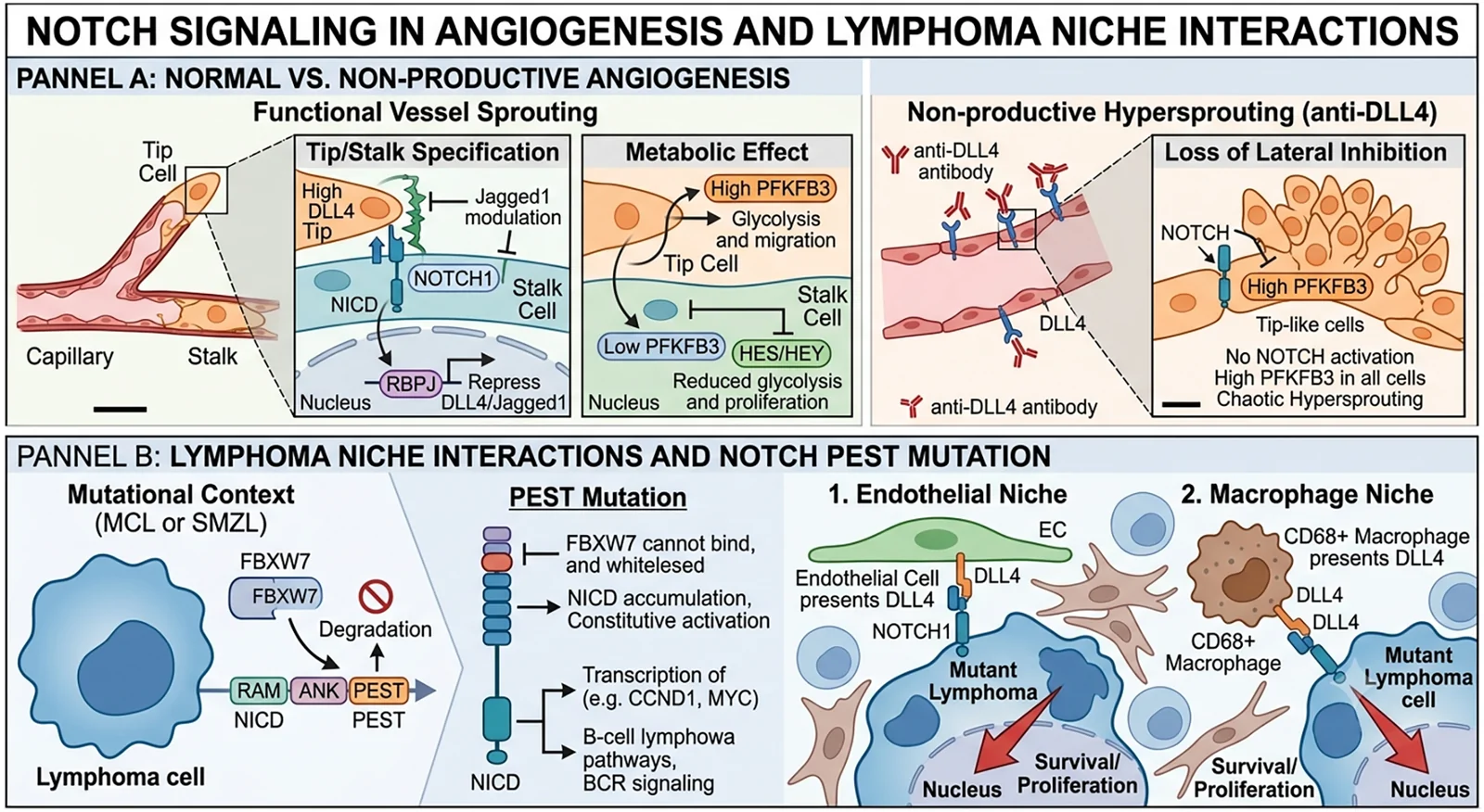
The role of NOTCH signaling in angiogenesis and lymphoma-microenvironment interactions. **(A)** Functional versus non-productive sprouting angiogenesis. **(A)** Angiogenesis & NOTCH Signaling. Functional Sprouting (Normal): Tip cells (high DLL4) activate NOTCH1 in adjacent Stalk cells. This lateral inhibition regulates endothelial metabolism (via PFKFB3), balancing healthy vessel growth. Chaotic Hypersprouting (Anti-DLL4): Blockade of DLL4 deactivates NOTCH. All cells adopt a tip-like phenotype (high PFKFB3), leading to excessive, disorganized, and non-functional vessel growth. **(B)** NOTCH Hyperactivation in Lymphomas (MCL & SMZL). PEST Mutation: Normally, the NICD protein is targeted for degradation by FBXW7 via its PEST domain. In mutated lymphomas, the loss of this domain prevents degradation, leading to constitutive NICD accumulation. Niche Interactions: Endothelial cells and macrophages in the tumor microenvironment present the DLL4 ligand to the mutant lymphoma cells. The resulting NOTCH1 activation drives oncogenic targets (MYC, CCND1) and synergizes with BCR signaling to promote tumor survival and proliferation.

Endothelial cells are highly glycolytic, generating over 85% of their ATP through the breakdown of glucose rather than oxidative phosphorylation, an adaptation allowing survival in hypoxic environments. Unconstrained glycolysis can destabilize vessel structures. Notch signaling plays a central metabolic role by directly lowering the expression of (6-phosphofructo-2-kinase/fructose-2-phosphatase 3) (Pfkb3), a key rate-limiting driver of glycolytic flux. By downregulating Pfkfb3, Notch activity limits glycolysis within stalk cells, encouraging them to transition from a highly active sprouting phase into a quiescent, metabolically stable, mature vascular structure (14) (**Figure 3A**).

### Notch in B-cell malignancies

Notch signaling acts as a direct driving oncogene within the malignant cell compartment of numerous B-cell lymphoproliferative disorders. The pathway is frequently hyperactivated within neoplastic cells and cancer stem cells (CSCs), promoting cell survival, chemoresistance, and self-renewal (15, 16). Targeting Notch offers a dual clinical advantage: disrupting the supportive tumor vasculature while simultaneously exerting direct anti-proliferative effects on the malignant cells (17, 18). The mechanistic links between endothelial Notch signaling and NOTCH mutations in malignant B cells are fundamentally driven by microenvironmental crosstalk and synergistic pathway hyperactivation. The interaction operates through the following interconnected mechanisms: Ligand-Receptor Crosstalk: Vascular endothelial cells within the tumor microenvironment highly express Notch ligands like JAG1. When malignant B cells interact with the endothelium, these ligands trigger the activation of wild-type Notch receptors on the B cells (42). Synergy with genetic mutations: B cells frequently harbor gain-of-function NOTCH1 or NOTCH2 mutations (often truncations in the PEST domain). While mutations allow for constitutive, ligand-independent signaling, endothelial-derived ligand activation provides an additional layer of stimulation. This dual activation hyper-drives downstream oncogenic networks (43). Downstream Oncogenic Convergence: Both intrinsic genetic mutations and endothelial mediated signaling converge to stabilize the Notch intracellular domain (NICD). This accumulation drives the transcription of key survival and proliferative genes, most notably MYC, and upregulates anti-apoptotic proteins (29). Angiogenesis and Extravasation: In return, factors secreted by malignant B cells can aberrantly activate endothelial Notch signaling, driving pathological angiogenesis. This process structurally modifies the vascular niche, allowing aggressive B cells to easily cross endothelial barriers and infiltrate extramedullary tissues (e.g., lymph nodes) (44).

Activating somatic mutations in NOTCH1 and NOTCH2 represent the most frequent secondary genetic alterations in mantle cell lymphoma (MCL), detected in 5–10% of clinical cases. These mutations delineate a highly aggressive clinical subset characterized by significantly shortened overall survival (OS) rates and a strong correlation with pleomorphic and blastoid cytological variants, which are resistant to standard immunochemotherapy regimens. Genomic analysis indicates that over 85% of these mutations map directly to the highly conserved C-terminal PEST domain of the NOTCH1 and NOTCH2 proteins. In wild-type receptors, the PEST domain regulates protein stability, serving as a specific recognition site for E3 ubiquitin ligases (such as FBXW7) that target the active nuclear NICD for proteasomal degradation, keeping the signal transient (19–21) (**Figure 3B**).

Somatic mutations (typically nonsense or frame-shift mutations) truncate or delete the PEST domain, preventing proper ubiquitin-mediated degradation. This results in prolonged intracellular accumulation of NICD, leading to sustained, constitutive transcriptional hyperactivation of downstream oncogenic targets. In addition to cell-intrinsic genetic mutations, MCL cells are sustained by extrinsic stimulation from the lymph node microenvironment (19, 20, 22). The ligand DLL4 is highly expressed within the vascular endothelium and by specific CD68-positive tumor-associated macrophages in the MCL nodal compartment. This expression constructs a specialized protective niche (23). Sustained engagement of Notch receptors on lymphoma cells by microenvironmental DLL4 enhances cell migration, tissue invasion, and paracrine pro-survival signaling (24). *In vitro* studies have demonstrated that this microenvironment-driven stimulation can be blocked using a humanized monoclonal anti-Notch1 antibody, highlighting its potential as a therapeutic target (25–27).

Extranodal marginal zone lymphoma of MALT lymphoma arises within chronic inflammatory or autoimmune sites (28, 29). Somatic mutations altering NOTCH1 and NOTCH2 are present in roughly 8% of unselected MALT lymphoma cases. Next-generation sequencing (NGS) analysis identified Notch1 alterations in 3 out of 5 exploratory MALT cases, pointing to pathway involvement (30–32).

In Diffuse Large B-Cell Lymphoma (DLBCL) cohorts, elevated expression rates of NOTCH1 correlate with aggressive clinical parameters (33). NOTCH1 mutations in DLBCL exhibit a distinct clinical correlation with chronic viral infection. Comparative screening identified somatic NOTCH1 mutations in 4% of DLBCL patients positive for the Hepatitis C Virus (HCV^+^), whereas these mutations were absent in HCV-negative cohorts. In HCV^+^ patients, mutated NOTCH1 correlates with a reduced survival rate, serving as an independent prognostic indicator of rapid malignancy advancement (34, 35).

Somatic activating mutations in NOTCH1 (frequently targeting the PEST domain) occur in approximately 5% of Splenic Marginal Zone Lymphoma (SMZL) cases (36). These mutations represent an important recurring clonal driver that disrupts physiological marginal zone B-cell differentiation, driving neoplastic proliferation and serving as a molecular marker for a higher risk of transformation into high-grade lymphoma (31).

Notch signaling may operate within distinct vascular and stromal niches, including lymph nodes, bone marrow, spleen, and extranodal tissues. In bone marrow, Notch signaling governs the balance between hematopoietic stem cells (HSCs) self-renewal and differentiation. Stromal cells and vascular endothelial cells within the bone marrow express Notch ligands like Jagged1 and Dll (37). Perisinusoidal endothelial cells utilize Dll4 to maintain HSC regenerative capacity. Signaling intensity dictates cell fate; intermediate Notch signals are conducive to hematopoietic commitment, whereas aberrations can lead to myeloid skewing or hematological malignancies (38). In the lymph nodes, fibroblastic reticular cells (FRCs) and follicular dendritic cells (FDCs) use Notch ligands to interact with migrating T and B lymphocytes. This crosstalk is critical for the architectural maintenance of lymphoid conduits and survival signals required during T-cell and B-cell maturation (39) In the spleen, Notch signaling (particularly via Notch2) is necessary for the development and maintenance of marginal zone B cells and specialized dendritic cells involved in rapid blood-borne antigen presentation (40).

In the extranodal tissues, during chronic inflammation or tumorigenesis, vascular endothelial cells in non-lymphoid tissues upregulate Notch ligands. This can promote pathological angiogenesis and facilitate the recruitment and survival of immune cells or support the formation of tertiary lymphoid structures (TLSs) (41).

It is important to underline the distinction between ligand-dependent and mutation-driven Notch activation in malignant B cells. The DLL4-mediated vascular/stromal niche model describes a pathogenic crosstalk in which DLL4 expressed by microenvironmental cells (like vascular endothelial cells) hyperactivates Notch signaling in malignant B cells. This pathway is especially prominent and aggressive in cases with NOTCH1 mutations. DLL4 is highly expressed in the lymph node vascular endothelium and by CD68+ macrophages/histiocytes. Malignant cells in MCL and CLL express Notch1. In NOTCH1-mutated disease, structural mutations lead to impaired receptor degradation. This causes an accumulation of the active Notch intracellular domain (NICD) in the nucleus (42). By comparison, the functional consequences of NOTCH2 mutations in SMZL, MALT lymphoma, and HCV-associated DLBCL remain less well defined.

Notch signaling is heavily dictated by specific mutational hotspots that drive aberrant receptor cleavage, enhance pathway activation, and fuel oncogenic hallmarks. The mutational landscape primarily targets two key domains across the NOTCH1–4 genes. Recurrent mutations in the heterodimerization domain (HD) destabilize the negative regulatory region. This causes ligand-independent cleavage, allowing gamma-secretase to constitutively liberate the Notch Intracellular Domain (NICD) (43). Truncating mutations, frameshifts, and nonsense mutations cluster frequently in the PEST domain. The PEST domain is responsible for marking the NICD for rapid ubiquitin-mediated degradation. Mutations here remove this sequence, leading to a drastically prolonged half-life of active NICD in the nucleus (44). When these structural and regulatory hotspots are altered, the downstream signaling output shifts from a tightly controlled developmental mechanism to continuous, oncogenic transcription.

### The notch-mediated lymphoid niche and immune microenvironment

The pathophysiological relevance of Notch signaling in B-cell malignancies extends beyond cell-intrinsic oncogenic mutations, operating as a bidirectional signaling hub within the tumor microenvironment (TME). In the lymphoma niche, a malignant positive-feedback loop is established between neoplastic B-cells, endothelial cells, and immune infiltrates, deeply affecting the functional state of both innate and adaptive immune cell subpopulations (45). The innate immune compartment of the lymphoma niche is heavily dominated by tumor-associated macrophages (TAMs) and myeloid-derived suppressor cells (MDSCs) (46).

Macrophage polarization is profoundly influenced by Notch signaling within the lymphoma microenvironment, resulting in the predominance of tumor-associated macrophages (TAMs) with an M2-like phenotype rather than tumoricidal activity. Hypoxic signals within the niche drive these CD68^+^ cells to overexpress DSL ligands (DLL1, DLL4, and Jagged1). Upon engaging Notch receptors on neighboring malignant B cells, these ligands activate a survival program characterized by the upregulation of anti-apoptotic proteins (Bcl-2, Mcl-1) and chemokine receptors (CXCR4, CCR7), ultimately promoting homing and tissue invasion. At the same time, Notch activation within monocytes favors their differentiation into immunosuppressive M2 macrophages instead of pro-inflammatory M1 cells, thereby reinforcing a chronic pro-tumoral feedback loop (45, 46). The comprehensive landscape of these somatic mutations across different B-cell malignancies and their respective microenvironmental impacts is summarized in **Table 1**.

**Table 1:** Landscape of NOTCH alterations in B-cell malignancies and the TME.

Malignancy/compartment	Frequency of mutation	Primary target domain/mechanism	Clinical & prognostic impact
Mantle cell lymphoma (MCL)	5–10%	C-terminal PEST domain of NOTCH1 and NOTCH2 (nonsense/frameshift)	Aggressive clinical subset, blastoid/pleomorphic variants, poor overall survival (OS), chemoresistance.
Splenic marginal zone lymphoma (SMZL)	~5%	NOTCH1 PEST domain mutations; occasional NOTCH2 alterations	Recurring clonal driver, disruption of marginal zone B-cell differentiation, higher risk of high-grade transformation.
MALT lymphoma	~8%	NOTCH1 and NOTCH2 somatic alterations	Associated with chronic inflammatory/autoimmune sites; potential driver in advanced/refractory cases.
Diffuse large B-cell lymphoma (DLBCL)	4–8%	NOTCH1 mutations; tightly correlated with HCV+ status	Independent prognostic indicator of rapid disease progression and reduced survival in HCV-positive cohorts.
Tumor microenvironment (TAMs, MDSCs, Tregs)	Non-mutational/Microenvironmental	Ligand over-expression (DLL4/Jag1) and RBPJ-mediated immune suppression	Epigenetic and hypoxic reprogramming leads to T-cell exhaustion (PD-1+) and immune evasion.

The immunosuppressive activity of myeloid-derived suppressor cells (MDSCs) is further amplified by Notch pathway activation through the RBPJ-dependent cascade. In these cells, Notch signaling induces the expression of arginase-1 (Arg-1) and inducible nitric oxide synthase (iNOS), enzymes responsible for depleting essential amino acids, such as L-arginine, from the extracellular space. This metabolic reprogramming effectively inhibits local T-cell proliferation and activation, thereby strengthening tumor-induced immune suppression (47, 48).

Adaptive T-cell subpopulations are profoundly reshaped by altered Notch signaling within the lymph node and splenic microenvironment, resulting in a marked shift toward immune tolerance. Among these populations, cytotoxic CD8^+^ T lymphocytes (CTLs) are particularly affected. Although physiological Notch1 signaling is required for their differentiation and effector function, chronic exposure to elevated levels of Notch ligands in the immunosuppressive tumor microenvironment (TME) promotes a dysfunctional state. Persistent Notch activation correlates with the increased expression of inhibitory immune checkpoint receptors, including Programmed Cell Death Protein 1 (PD-1), Cytotoxic T-Lymphocyte-Associated Protein 4 (CTLA-4), and T-cell Immunoglobulin and Mucin-Domain Containing-3 (TIM-3). Consequently, CTLs acquire an exhausted phenotype that severely compromises their ability to mediate an effective anti-lymphoma cytotoxic response (49).

Regulatory T cells (Tregs), characterized by the CD4^+^ CD25^+^ Foxp3^+^ phenotype, are co-opted by the tumor, which actively promotes their expansion through the Notch–RBPJ axis. Notch signaling directly cooperates with Transforming Growth Factor-beta (TGF-β) pathways to stabilize Foxp3 expression in naïve CD4^+^ T cells, converting them into functional, suppressive Tregs. These cells actively shield the neoplastic B cells from residual immune surveillance by secreting high levels of Interleukin-10 (IL-10) and TGF-β (49).

The dendritic cell (DC) compartment, which is responsible for antigen presentation and the initiation of adaptive immune responses, is similarly impaired by the deregulation of this pathway. Notch signaling (particularly via the DLL1 ligand) is normally essential for the maturation of conventional dendritic cells (cDCs). However, in the presence of tumor-derived factors and Jagged1-mediated competitive inhibition, DC maturation is arrested. These immature DCs exhibit significantly lower levels of Major Histocompatibility Complex class II (MHC-II) and co-stimulatory molecules (CD80, CD86), making them incompetent in cross-presenting lymphoma-associated antigens to naïve T cells, thereby preventing the initiation of a systemic anti-tumor immune response (50).

#### Tumor angiogenesis and therapeutic targeting notch signaling

Tumor blood arteries and xenografts frequently exhibit marked upregulation of Notch1 activity alongside hyper-expression of ligands DLL4 and Jag1, driven by constant hypoxia and oncogenic signals. The reliance of tumor vessels on the DLL4-Notch lateral inhibition axis has made this pathway a prime target for anti-angiogenic strategies. Pharmacological disruption of this pathway by means of monoclonal antibodies against DLL4 or Notch1, or systemic γ-secretase inhibitors (GSIs)-produces a unique, highly reproducible physiological phenomenon known as non-productive hypersprouting (51, 52).

The Loss of Lateral Inhibition consists in Inhibiting the Notch pathway that removes the regulatory brakes on neighboring endothelial cells. Deprived of Notch1 signaling, adjacent stalk cells immediately upregulate VEGFR2/3, regain high responsiveness to VEGF-A (11).

Chaotic Network Formation: this widespread transformation causes unconstrained, chaotic vascular sprouting (53). Endothelial cells sprout in all directions, creating an excessively dense, tangled, and highly disorganized vascular plexus. Perfusion Failure and Necrosis: This hyper-branched network is structurally defective, lacking functional lumens, coherent directionality, proper pericyte coverage, or coordinated blood flow. Consequently, despite the massive increase in total vessel count, functional tumor perfusion drops to near zero. This total vascular failure starves the neoplastic tissue of oxygen and nutrients, culminating in extensive central tumor necrosis (54–56).

#### Vessel normalization

Rather than destroying tumor vessels, this approach aims to therapeutically activate endothelial Notch signaling to correct the structural defects of the tumor vasculature. Re-activating Notch can downregulate VEGFRs expression, prune redundant vessel branches, tighten endothelial junctions, and recruit pericytes (57). Leaky, tortuous tumor vessels are transformed into mature, functional conduits, alleviating tumor hypoxia and markedly enhancing the delivery of co-administered chemotherapeutic drugs or radiation therapies (13). Different notch inhibitors, including monoclonal antibodies, decoy receptors, GSIs, are undergoing Phase I/II clinical trials, but there are currently no clinically approved pharmaceutical agents capable of selectively activating the Notch cascade (7, 58).

Preclinical models for both MCL and CLL have shown that specific anti-Notch1 monoclonal antibodies (such as OMP-52M51) and GSIs can block this pathogenic signaling, reversing tumor proliferation and migration (59).

Therapeutic inhibition of Notch signaling in B-cell malignancies translates into chemosensitization, disruption of the tumor microenvironment, and targeted apoptosis of malignant cells. Emerging strategies are shifting from broad inhibition to receptor-specific therapies to enhance clinical efficacy while mitigating off-target toxicities. Sensitization to Chemotherapy: Notch inhibition (e.g., via γ-secretase inhibitors or GSIs) in B-cell Acute Lymphoblastic Leukemia (B-ALL) increases intracellular reactive oxygen species (ROS) levels. This enhances blast cell apoptosis when Notch blockers are combined with conventional chemotherapeutic agents (60). B-cell malignancies, including multiple myeloma and CLL, heavily rely on paracrine Notch signaling with bone marrow stromal cells for survival signals. Blocking these pathways deprives the cancer stem cells of their protective niche, leading to growth arrest (61). First-generation pan-Notch inhibitors (GSIs) often caused severe gastrointestinal toxicities by non-specifically blocking Notch receptors vital for normal gut homeostasis. Translational focus is now directed toward isoform-specific monoclonal antibodies (targeting only the NOTCH2/NOTCH3 receptors highly expressed in B-cells) and ADAM protease inhibitors (62). Recent studies indicate that Notch pathway blockade helps reshape the tumor microenvironment. It facilitates reduced macrophage infiltration and enhanced CD8^+^ T-cell activation, paving the way for combining Notch inhibitors with immune checkpoint blockade (ICB) or CAR T-cell therapies (63).

### Translational challenges and resistance mechanisms

Despite the strong rationale for targeting the Notch cascade in B-cell lymphoproliferative disorders and tumor angiogenesis, clinical translation has faced severe bottlenecks. The primary obstacle for systemic pan-Notch inhibitors, such as first-generation GSIs, is severe, dose-limiting gastrointestinal toxicity (64). Because Notch1 and Notch2 are essential for maintaining crypt progenitor cell differentiation in the gut, their simultaneous blockade induces rapid secretory metaplasia, characterized by the massive conversion of proliferative crypt cells into goblet cells, leading to debilitating diarrhea (65). To circumvent this, current strategies have shifted toward monoclonal antibodies (mAbs) targeting specific receptors (e.g., anti-Notch1 or anti-Notch2) or ligands (e.g., demcizumab against DLL4). However, even target-specific inhibition introduces unique challenges (66). While anti-DLL4 therapy successfully induces non-productive hypersprouting and tumor necrosis, prolonged clinical administration has been associated with severe vascular toxicities, including subcutaneous hemangiomas, congestive heart failure, and pathological endothelial proliferation driven by the chronic disruption of physiological lateral inhibition in healthy organs (67). An overview of the current pharmacological classes targeting the Notch cascade, including their representative compounds and clinical limitations, is provided in **Table 2**.

**Table 2:** Notch-targeted therapeutic strategies and clinical status.

Agent class	Representative compounds	Target/Mechanism	Current clinical status & limitations
Pan-notch inhibitors (GSIs)	Nirogacestat, AL101	Block γ-secretase cleavage, preventing NICD release	High gastrointestinal toxicity (secretory metaplasia); requires strict intermittent dosing schedules.
Monoclonal antibodies (mAbs)	Brontictuzumab (anti-Notch1), Demcizumab (anti-DLL4)	Selective inhibition of specific receptors or ligands	Reduced systemic toxicity compared to GSIs; risks of vascular complications (hemangiomas) with anti-DLL4.
Decoy receptors & traps	DLL4-Fc, Notch1-EGF repeats	Soluble fusion proteins competing for ligand/receptor binding	Preclinical and early Phase I evaluations; variable bioavailability within dense tumor extracellular matrices.
Pan-notch inhibitors (GSIs)	Nirogacestat, AL101	Block γ-secretase cleavage, preventing NICD release	High gastrointestinal toxicity (secretory metaplasia); requires strict intermittent dosing schedules.
Selective activators (normalization)	Engineered agonistic mAbs	Target specific receptor domains to enforce lateral inhibition	Early experimental stage; no clinically approved agents available yet, difficult to titrate to avoid over-activation.

Malignant cells and the surrounding stroma rapidly develop resistance to Notch-targeted therapies through several mechanisms that circumvent therapeutic blockade. One of the most common involves loss-of-function mutations in negative regulators of the pathway, particularly the E3 ubiquitin ligase FBXW7. Secondary mutations or deletions affecting FBXW7 prevent the degradation of even wild-type NICD, thereby sustaining nuclear transcriptional activity and effectively bypassing upstream pharmacological inhibition mediated by γ-secretase inhibitors (GSIs) (68). Simultaneously, malignant B cells can escape therapy through the activation of non-canonical Notch signaling. Instead of relying on canonical RBPJ-dependent transcription, membrane-bound Notch receptors directly engage alternative signaling pathways, including the PI3K/Akt/mTOR and NF-κB cascades, preserving survival and anti-apoptotic programs despite inhibition of the core pathway (69). Furthermore, adaptive changes within the tumor microenvironment contribute to therapeutic resistance. In response to selective anti-DLL4 pressure, stromal cells frequently upregulate Jagged1 expression, which acts as a competitive antagonist and reduces residual therapeutic efficacy while maintaining stalk-cell plasticity and alternative vascular survival networks (70).

## Concluding remarks

The detailed characterization of the genetic, epigenetic, and microenvironmental factors governing the Notch receptor-ligand network has improved our understanding of vascular compartment and hematological oncology. Due to the differential physiological roles of individual receptor isoforms, such as Notch2 in marginal zone B-cell development and Notch1 in T-cell commitment and endothelial patterning, dissecting these pathways provides crucial insight into tissue-specific oncogenesis. Incorporating routine, high-efficiency screening for NOTCH1 and NOTCH2 aberrations (such as PEST domain truncations) alongside co-occurring mutations like KMT2D into clinical diagnostic panels represent a valuable prognostic factor. These molecular markers can optimize diagnostic accuracy, refine survival risk stratification, and guide surveillance in poor-prognosis lymphoma subtypes.

Finally, these insights lay the necessary biological groundwork for the precise, personalized deployment of selective Notch pathway inhibitors, decoy formulations, and anti-angiogenic strategies to overcome chemoresistance and improve outcomes in refractory hematological diseases.
